# Altered Corticomuscular Coherence (CMCoh) Pattern in the Upper Limb During Finger Movements After Stroke

**DOI:** 10.3389/fneur.2020.00410

**Published:** 2020-05-14

**Authors:** Ziqi Guo, Qiuyang Qian, Kiufung Wong, Hanlin Zhu, Yanhuan Huang, Xiaoling Hu, Yongping Zheng

**Affiliations:** Department of Biomedical Engineering, The Hong Kong Polytechnic University, Kowloon, Hong Kong

**Keywords:** stroke, compensatory contraction, upper limb, corticomuscular coherence (CMCoh), finger motion

## Abstract

**Background:** Proximal compensation to the distal movements is commonly observed in the affected upper extremity (UE) of patients with chronic stroke. However, the cortical origin of this compensation has not been well-understood. In this study, corticomuscular coherence (CMCoh) and electromyography (EMG) analysis were adopted to investigate the corticomuscular coordinating pattern of proximal UE compensatory activities when conducting distal UE movements in chronic stroke.

**Method:** Fourteen chronic stroke subjects and 10 age-matched unimpaired controls conducted isometric finger extensions and flexions at 20 and 40% of maximal voluntary contractions. Electroencephalogram (EEG) data were recorded from the sensorimotor area and EMG signals were captured from extensor digitorum (ED), flexor digitorum (FD), triceps brachii (TRI), and biceps brachii (BIC) to investigate the CMCoh peak values in the Beta band. EMG parameters, i.e., the EMG activation level and co-contraction index (CI), were analyzed to evaluate the compensatory muscular patterns in the upper limb.

**Result:** The peak CMCoh with statistical significance (*P* < 0.05) was found shifted from the ipsilesional side to the contralesional side in the proximal UE muscles, while to the central regions in the distal UE muscle in chronic strokes. Significant differences (*P* < 0.05) were observed in both peak ED and FD CMCohs during finger extensions between the two groups. The unimpaired controls exhibited significant intragroup differences between 20 and 40% levels in extensions for peak ED and FD CMCohs (*P* < 0.05). The stroke subjects showed significant differences in peak TRI and BIC CMCohs (*P* < 0.01). No significant inter- or intra-group difference was observed in peak CMCoh during finger flexions. EMG parameters showed higher EMG activation levels in TRI and BIC muscles (*P* < 0.05), and higher CI values in the muscle pairs involving TRI and BIC during all the extension and flexion tasks in the stroke group than those in the control group (*P* < 0.05).

**Conclusion:** The post-stroke proximal muscular compensations from the elbow to the finger movements were cortically originated, with the center mainly located in the contralesional hemisphere.

## Introduction

Post-stroke motor recovery is usually associated with the cortical reorganization and adaptive learning experiences (1). Cerebral plasticity is the process by which the human body reorganizes neural networks and pathways after a stroke. Existing studies have found that the majority of motor recovery observed via cerebral plasticity reaches a plateau within the first 6 months after the onset (2, 3). Patients with chronic stroke (first onset over 6 months) regain the independence of the activities of daily living but always sustain upper extremity (UE) motor dysfunctions, e.g., muscle weakness, spasticity, and discoordination (4). Specifically, patients' distal UE segments, e.g., fingers and wrist, usually exhibit poorer functional recovery than the proximal elbow and shoulder parts (5). In our previous study (6, 7), we found that the dyscoordination observed following chronic stroke was particularly evident during distal UE joint motion tasks, and that stroke patients frequently relied on compensatory contractions from proximal UE muscles to substitute for a loss or reduction in hand function. However, Jones concluded that proximal compensations can be mistaken for recovery and constrain the potential motor restoration at the distal segments, leading to “learned non-use” or “learned dis-use” (8). Although such post-stroke behavioral deviation can further exacerbate motor impairments, the interaction between the cortical plasticity in chronic stroke and the dynamic muscular coordination in the upper limb has not yet been well-investigated.

Previous neuroimaging studies on motor restoration after stroke using positron emission tomography (PET), functional magnetic resonance (fMRI) imaging, and transcranial magnetic stimulation (TMS) have identified that post-stroke patients exhibit a reduction in brain activities at the lesioned side and a propensity to recruit the contralesional motor cortex when conducting tasks involving the arms (9–11). However, these methods were limited by the low temporal resolutions to reveal the transient relationship between the cortical and muscular dynamics in the investigation of the post-stroke compensatory mechanism to activate proximal muscle contractions in compensation for distal movements in the upper limb.

Electroencephalogram (EEG) and electromyogram (EMG) can capture faster dynamics in the cortex and peripheral muscles, respectively, comparing with the imaging techniques mentioned above. Furthermore, previous studies have found that the coherence between the two parameters can result in the demonstration of time-based functional connections in the neuromuscular pathways when subjects perform specific motion tasks (12, 13). This also makes it possible to identify the location of cortical sources and trace the neuroplasticity after stroke according to the coherence topography (14). The coherence between EEG and EMG was first described by Salenius et al. (15) and Gerloff et al. (16), who referred to it as corticomuscular coherence (CMCoh) to reflect voluntary descending control from the primary motor cortex to the effector muscles. Coherence can be calculated using both EEG and EMG signals, and it is typically observed within the frequency range of 13–30 Hz (Beta band) during the execution of steady-state isometric contraction and phasic movements (17). The maximum value (i.e., the peak CMCoh) denotes the most significant neuromuscular coupling of the coherent activities and location of the central generator over the whole motor cortex (18, 19). Mima et al. (20) first reported the topographical shift of CMCoh from the lesional side to the contralesional side observed among chronic stroke patients, which may be due to the contribution of lateral and/or medial premotor area control made to the muscles, as suggested by previous PET and electrocorticographic studies (21–23). Furthermore, the neuromuscular coupling between cortical commands and consequent muscle activities indicated by CMCoh values is usually not evident immediately after a stroke; rather, it seems to increase throughout the course of the recovery process gradually. Fang et al. (24) and Larsen et al. (25) reported that the CMCoh values in patients with acute and subacute stroke were weaker than those observed in unimpaired controls, while Chen and colleagues (26) found patients with chronic stroke demonstrated higher CMCoh values from the UE flexors than those in a control group. These studies have consistently indicated that data pertaining to the intensity and location of peak coherence could be employed to estimate the muscle representation areas after neural reorganization following stroke. However, most of the CMCoh studies on stroke patients to date investigating the cerebral-derived control on distal UE segments have been limited to EMG recording from distal muscles, e.g., the extensor carpi radialis muscle (19) or its antagonist muscle flexor carpi radialis (27) in wrist extension at the affected side. Rare studies have employed CMCoh to investigate the contractions of proximal muscles to compensate for distal motions, which could be traced back to a cortical-originated alteration in muscular discoordination at the peripheral.

The purpose of this study was to investigate the corticomuscular coordination pattern in the upper limb muscles during distal finger movements at the affected side of patients with chronic stroke, via a combination of EEG and EMG measurements.

## Materials and Methods

In the current study, we analyzed the CMCoh of both chronic stroke subjects and age-matched unimpaired subjects to make comparisons on their coherent activities between the motor cortex and effector muscles. The EMG parameters were also analyzed to evaluate the peripheral muscular coordination across the proximal and distal UE segments. For the stroke subjects, the experiment was performed on the affected hand, while the habitual hand was investigated in the case of the unimpaired subjects.

### Experimental Setup

#### EEG and EMG Electrode Configuration

**Figure 1:**
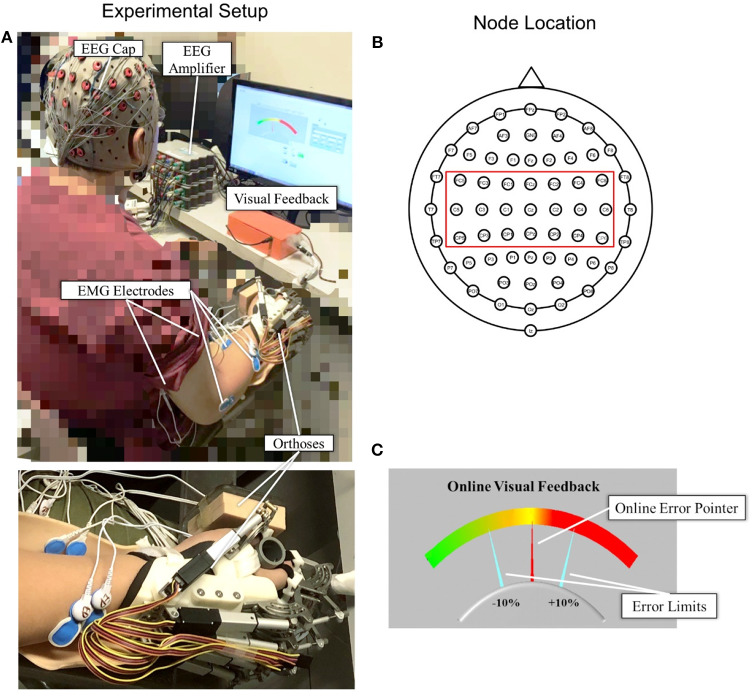
**(A)** The experimental setup with an enlarged photo of the forearm and hand configuration; **(B)** top view of the 64-channel g.GAMMAsys cap with 21 channels selected for CMCoh analysis; **(C)** the interface of visual feedback for real-time contraction level control: The fixed yellow midline is the target contraction level, and the red pointer shows the real-time muscular contraction level.

Figure 1A shows the experimental setup of this study. Each subject was comfortably seated in a chair in front of a 14-inch computer screen with his or her testing forearm placed in the neutral position on a horizontally fixed slab. A robotic hand orthosis consisting of a palm-wrist module and five individual finger assemblies (6, 7) was used to fix the wrist straight at a 0° angle. The index, middle, ring, and little fingers of the subjects were fixed at a position of 135° at the metacarpophalangeal (MCP) joint and 135° at the proximal interphalangeal (PIP) joints, which was at 50% open of the robotic hand orthosis, also with the thumb finger fixed at an angle of 180° at the MCP joint and 165° at the PIP joint. Then, the experimental upper limb of a subject was attached to the palm orthosis on the slab after wearing the robotic hand, as shown in Figure 1A. The weight of the robotic hand is 500 g. The patient arm after wearing the robotic hand was gravity compensated with this setting during the whole experiment. A 64-channel g.GAMMAsys active electrode EEG system referenced to left earlobe and ground at *AF*_*z*_ was mounted on the subject's scalp according to the 10-20 system. The EEG signals from the 21 channels (i.e., C_Z_, C_1_, C_2_, C_3_, C_4_, C_5_, C_6_, CP_Z_, CP_1_, CP_2_, CP_3_, CP_4_, CP_5_, CP_6_, FC_Z_, FC_1_, FC_2_, FC_3_, FC_4_, FC_5_, FC_6_, as shown in Figure 1B) covering the sensorimotor area were adopted for the CMCoh analysis. All EEG signals were amplified 10,000 times (amplifier: g.USBamp, USA) before being band-pass filtered from 1 to 100 Hz with a 50 Hz Notch filter. EMG signals were collected from the antagonist muscle pair for finger extension/flexion, i.e., extensor digitorum (ED) and flexor digitorum (FD), and the antagonist muscle pair for elbow extension/flexion, i.e., triceps brachii (TRI) and biceps brachii (BIC), of subjects' UEs. Four pairs of EMG electrodes were attached to the skin surface of the four muscle bellies, with a 2-cm center separation. The reference electrode was attached to the surface of the olecranon. All EMG signals were amplified with a gain of 1000 (amplifier: INA 333, Texas Instruments Inc.) and filtered by a 10 to 500 Hz band-pass filter and the 50 Hz notch filter (7, 28). The impedances of all EEG and EMG electrodes were maintained below 5 kΩ. Both the EEG and EMG signals were synchronized with a sampling frequency of 1,200 Hz by a DAQ card (NI, USB-6009 14-Bit Multifunction DAQ USB) and stored for offline processing. Square wave markers (0.5 s in duration, 2 V for the start and 1 V for the end of the recording) were denoted onto the EEG and EMG trials for synchronized timing in the same recording. Furthermore, the EMG signals produced by the ED and FD were also used for later online processing in a visual feedback motion control evaluation of isometric finger contractions.

#### Visual Feedback Interface

A self-programmed operation interface based on LABVIEW 2015 was used for visual feedback motion control during the experiment, as shown in Figure 1C. The background panel of the screen exhibited a color gradient from left to right, which indicated the variation of contraction level calculated from the real-time EMG of a target muscle. Two fixed aquamarine pointers denoted the acceptable ±10% of the target contraction level with an indicated range in the visual feedback during the dynamic control, as per the work of Meng et al. (19). The real-time contraction level indicated by EMG was calculated as follows:

(1) $$\begin{array}{l} {EMG}_{c(ED\;orFD)}=\frac{{EMG}_{(ED\;or\;FD)}-{EMG}_{base}}{{EMG}_{\max(ED\;or\;FD)}-{EMG}_{base}}\times 100\% \end{array}$$

where ***EMG***_**(*EDorFD*)**_was the mean of the absolute real-time EMG envelope of the ED or FD muscle (i.e., rectified EMG with 10 Hz low-pass filtering) in a 100 ms windows under finger extension or flexion motion task; ***EMG***_***max*(*EDorFD*)**_ represented the average of the absolute EMG envelope value of the ED muscle in an isometric maximum voluntary extension (iMVE) or FD muscle in an isometric maximum voluntary flexion (iMVF) contraction conducted at the beginning of each experiment; ***EMG***_***base***_ was the average of the absolute EMG envelope of the muscle during resting state; ***EMG***_***c*(*EDorFD*)**_ represented the contraction level performed in response to the real-time visual feedback in the user interface (Figure 1C) at values ranging from 0 to 100% (0% represented resting status while 100% represented the maximal value from iMVE or iMVF). The isometric maximum voluntary contraction (iMVC) test would be introduced in the protocol later.

### Subject Recruitment

After obtaining approval from the Human Subjects Ethics Subcommittee of the Hong Kong Polytechnic University, chronic stroke survivors (stroke group) and age-matched unimpaired subjects (control group) were recruited and subsequently underwent EEG-EMG assessments in an electromagnetic shielded laboratory.

The inclusion criteria for subjects with chronic stroke were as follows: (1) aged between 35 and 70 years old; (2) had a diagnosed unilateral brain lesion due to stroke onset more than 1 year, without other neurological deficits or secondary onset (3, 7) had sufficient cognition (as measured by the Mini-Mental State Examination [MMSE>21]), to understand the content or purpose of the study and follow simple instructions during the assessment (4, 29) had a unilateral UE motor impairment that ranged from severe to moderate, as measured by the Fugl-Meyer Assessment for UE (15 < FMA-UE <45, with a maximal score of 66) (5, 30) had ≤ 3 level muscle tension at the elbow, wrist, and fingers at the time of enrollment, as assessed by the Modified Ashworth Scale (MAS) (6, 31) had detectable voluntary EMG (i.e., three times SD above the baseline) from four UE muscles, i.e., ED, FD, TRI, and BIC, within the affected arm (32). The inclusion criteria for the unimpaired subjects were as follows: (1) aged between 35 and 70 years old; (2) no history of any neurological deficits; (3) no upper limb motor dysfunction due to any kind of osteoarticular or peripheral neuromuscular disease. Furthermore, subjects who were pregnant, had been previously diagnosed with severe dysphasia or hypertension, or participated in any intensive upper limb physical practice or botulinum toxin treatment within 1 year before the current experiment were excluded from participating in the research. According to the previous studies (26, 33, 34), the gender factor did not significantly affect the CMCoh parameters. Hence, we had no specific requirement on the gender in the recruitment process.

The final study population consisted of 14 chronic stroke patients and 10 unimpaired subjects. Written consent was secured from each participant after they were informed about the research purpose and content. Table 1 shows the demographic data of all subjects in both groups and the clinical scores of the subjects in the chronic stroke group.

**Table 1A:** **(A)** Demographic data of the stroke and age-matched control groups.

**Group**	**No. of Participants**	**Gender Female/Male**	**Age**	**Stroke Type Hemorrhage/Ischemic**	**Experiment Side Right/Left**
Stroke	14	3/11	56.5 ± 9.5	7/7	7/7
Control	10	4/6	50.8 ± 15.8	/	7/3

**Table 1B:** Motor impairments of the stroke subjects measured by clinical scores.

**Onset years**	**MAS**			**FMA**		
	**Finger**	**Wrist**	**Elbow**	**Full Score**	**Wrist/Hand**	**Shoulder/Elbow**
8.1 ± 4.2	1.1 ± 0.8	1.4 ± 0.7	1.6 ± 0.6	33.6 ± 9.9	11.1 ± 3.2	22.5 ± 7.3

### Experiment Protocol

#### iMVC Test

Each subject was instructed to conduct the iMVC test at the beginning of the experiment as follows: (1) Remain relaxed for 5 s to record the resting EMG levels over three repetitions; (2) execute distal UE joint iMVC movements, i.e., the fingers iMVE and iMVF, with the robotic hand orthosis at 50% open for 5 s over three repetitions; (3) execute proximal UE joint iMVCs, i.e., elbow iMVE and iMVF, for three times, respectively, with the arm fixed by an elbow orthosis with the shoulder abducted at 70° and the elbow flexed at 90°, as per our previous study (28, 35). The participants were provided with a 5 min rest period between two consecutive MVCs to prevent muscle fatigue. The mean values for the EMG envelopes of the three iMVCs of each agonist muscle (i.e., ED for finger iMVE, FD for finger iMVF, TRI for elbow iMVE, and BIC for elbow iMVF) were adopted as the maximum EMG amplitudes for the related muscles, denoted by ***EMG***_***max*(*i*)**_. The average of the EMG envelopes of the three resting motions of each muscle was denoted by ***EMG***_***base*(*i*)**_. All the raw EMG data obtained from the iMVC test were recorded and stored for further offline processing at a later stage. The elbow orthosis was removed after the iMVC test, while the palm and robotic hand orthoses continued to be used in the following finger motion tasks, as shown in Figure 1A.

#### Isometric Finger Extension/Flexion Tasks

After the iMVC test, each individual was instructed to conduct finger extension and flexion motions according to the same limb configuration presented in Figure 1A at different contraction levels. According to previous studies (19, 36, 37), constant and moderate (contraction level <50%) muscle contraction can demonstrate the most pronounced CMCoh in the Beta band range without the subject suffering from significant muscle fatigue, with less spontaneous discharges of muscles during the contraction compared with higher levels. The potential residual spontaneous discharges in the recorded EMG were further reduced by the baseline removal in Equation 1. In this work, four contraction schemes at 20 and 40% contraction levels for both finger extension (Ex) and flexion (Fx) were adopted as the motion target for CMCoh investigation, denoted as 20, 40% Ex, 20%, and 40% Fx. A summary of the schemes is provided in Table 2.

**Table 2:** The target contraction levels for the CMCoh investigation.

**Schemes**	**Description**
20%Ex	Finger extension at 20% iMVE of ED
40%Ex	Finger extension at 40% iMVE of ED
20%Fx	Finger flexion at 20% iMVF of FD
40%Fx	Finger flexion at 40% iMVF of FD

Each subject randomly performed the four motion schemes in the experiment (Table 2). The subjects were provided with a visual instruction on the screen showing the name of the target scheme and subsequently performed isometric extension or flexion contractions with the fixed 50% opened robotic hand orthosis at an appropriate strength to try and maintain the position of the red pointer at the midline of the plate (i.e., the target scheme). The ideal control corresponded to a 0% deviation from the midline over 35 s, and the fluctuation needed to be maintained within the ±10% error region, which was the achievable range for stroke subjects in the preliminary study (19). Each motion scheme was repeated five times with a 2 min rest after two consecutive contractions to minimize the effect of fatigue. The EMG mean power frequency (MPF) was calculated offline and any data with 10% reduction in the MPF was treated as fatigue (38). No fatigue was detected during the trials. All subjects were also instructed to minimize the possible bite, eye blinking and body movement during the contraction.

### EEG EMG Processing

All subjects were instructed to conduct 35 s contraction in each trial, and we omitted the last 5 s during the offline processing, and each contraction repeated for 5 times. Then, we chopped the signal trial into epochs with a unit length of 1,024 point/epoch when the sampling frequency was 1,200 Hz (19, 39, 40). In the current study, non-rectified EMG signals were used for the CMCoh calculation to minimize frequency distortion as a result of the rectification (41). A total of 150 s of EEG and EMG signals were collected from each subject over the course of the five trials, following which 175 epochs were obtained, i.e. (1,200^*^30/1,024)^*^5, with respect to each scheme presented in Table 2 and subsequently used for the coherence estimation as follows (42):

(2) $$\begin{array}{l} CMCoh\;\left(\sigma \right)=\frac{|\;f_{xy}\left(\sigma \right){|}^{2}}{f_{xx}\left(\sigma \right)\cdot f_{yy}\left(\sigma \right)} \end{array}$$

where ***f***_***xx***_(σ) and ***f***_***yy***_(σ) represented the auto-spectrum of the selected EEG signal and EMG signal, respectively, and ***f***_***xy***_(σ) represented the cross-spectrum of EEG and EMG. The confidence level was calculated according to the following equation:

(3) $$\begin{array}{l} {CL}_{(\alpha \%)}=1-{(1-\frac{\alpha }{100})}^{\frac{1}{N-1}} \end{array}$$

(4) $$\begin{array}{l} N=\frac{Sampling\;Rate\times Data\;Length\times Trial\;Number}{Epoch\;Length} \end{array}$$

where α was the significance level (α was 95 in this study, corresponding to a *P* value of 0.05); ***N*** was the number of data epochs (***N*** =175 in this study); and ***CL***_(α%)_ (0.0170 in this study) represented the coherence confidential limit, above which the coherence was considered to be significant.

The peak CMCoh of a target muscle (i.e., ED, FD, TRI, or BIC) was utilized to describe the highest significant coherence (19, 37, 40) among the 21 EEG channels and an EMG channel in the Beta band (13–30 Hz) during the finger extension and flexion movements. CMCoh topology was used to find the most related cortical activation area of the subjects in both groups.

In this study, EMG parameters were also adopted to investigate the muscle activation level and co-contraction pattern during the finger movements, (1) normalized EMG activation level (35) of ED, FD, TRI and BIC muscles; and (2) co-contraction index (CI)(35) between a pair of muscle (i.e., ED-FD, ED-TRI, ED-BIC, FD-TRI, FD-BIC, and TRI-BIC).

The EMG signals of a muscle ***i*** (i.e., ED, FD, TRI or BIC) were firstly normalized with the resting and iMVC EMG data expressed previously in iMVC Test as follows:

(5) $$\begin{array}{l} {nEMG}_{i}=\frac{{EMG}_{i}-{EMG}_{base(i)}}{{EMG}_{max(i)}-{EMG}_{base(i)}} \end{array}$$

Then the normalized EMG activation level was processed as follows:

(6) $$\begin{array}{l} \bar{EMG}=\frac{1}{T}\int_{0}^{T}{nEMG}_{i}\left(t\right)dt \end{array}$$

where $\bar{EMG}$ was the muscle activation level of muscle *i*, ***nEMG***_***i***_(***t***) was the normalized EMG linear envelope of the muscle over the duration of five isometric contractions, and ***T*** was the length of the signal.

The CI between a pair of muscles could be expressed as:

(7) $$\begin{array}{l} CI=\frac{1}{T}\int_{0}^{T}A_{ij}\left(t\right)dt \end{array}$$

where ***A***_***ij***_(***t***) represented the overlapping activity of the normalized EMG linear envelopes (as per Equation 6) of a pair of muscles (*i* and *j*). The CI value was between 0 (no overlapping) to 1 (fully overlapping) as adopted in previous robot-assisted post-stroke training studies (6, 7, 35). Higher CI values indicated a more significant co-contraction observed within the muscle pair, and lower CI values suggested a separation in the muscle activation across the muscle.

### Statistical Analysis

The Lilliefors method was used to conduct normality tests on both the CMCoh values and the EMG parameters. The findings revealed that the data were normally distributed (*P* > 0.05). The demographic data of the stroke and unimpaired control groups were examined for baseline differences using the independent *t*-test and the Fisher exact test. Subjects in both groups did not differ significantly in terms of age, gender, and the side of the experimental hand (*P* > 0.05). The amplitudes of peak CMCoh and EMG parameters were first analyzed using the independent *t*-test to compare the intergroup differences under different motion schemes. Subsequently, a paired *t*-test was performed to investigate the intragroup variation of CMCoh and EMG parameters with respect to the factor of contraction level (i.e., 20 and 40%) for each muscle when conducting extension and flexion motions. The statistical significance was set at 0.05 in this study. All statistical calculations in the study were performed using SPSS 24.0 (2016). The levels of statistical significance were also indicated at 0.05, 0.01, and 0.001.

## Results

All the stroke and unimpaired subjects completed the four motion schemes at the finger joints.

### Corticomuscular Coherence

**Figure 2:**
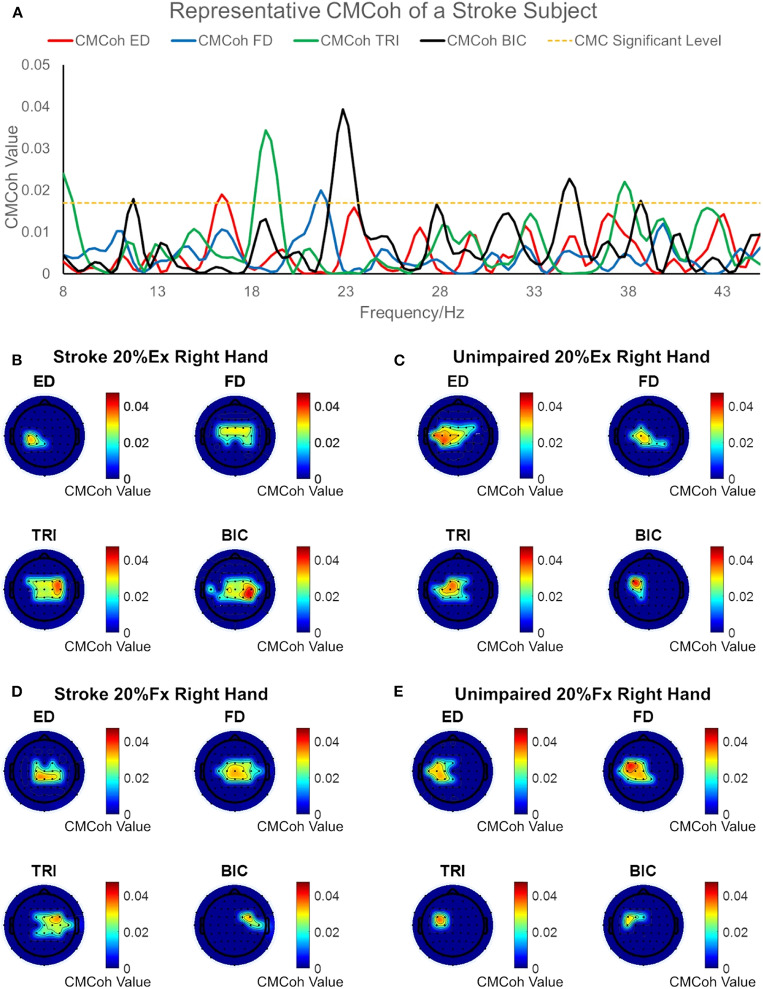
**(A)** Representative CMCoh spectra with CL (α% = 95%, *N* = 175) level from the peak CMCoh channels of a right hemiplegic subject in right hand 20% Ex; **(B–E)** representative topographical CMCoh map with peak CMCohs: **(B)** the stroke subject in right hand 20% Ex;**(C)** a right-handed unimpaired subject in right hand 20% Ex; **(D)** the stroke subject in right hand 20% Fx; **(E)** the unimpaired subject in right hand 20% Fx. The peak CMCoh values, corresponding frequency (Hz) and channels in **(B–E)** are shown in the table below.

Figure 2 illustrates the representative CMCoh spectra of the four muscles with corresponding frequencies to the peak value and representative topographic maps of coherence of a stroke subject and an unimpaired subject during right hand 20% Ex and 20% Fx schemes. When conducting 20% Ex, significant peak ED, FD, TRI, and BIC CMCohs were observed in the contralateral (left) hemisphere in the unimpaired subject. By contrast, the stroke subject presented peak TRI and BIC CMCohs on the contralesional (right) hemisphere and peak FD CMCohs on the central region showing the post-stroke shift of cortical activations during the 20% Ex scheme.When performing the 20% Fx movements, the pronouced shift of the peak CMCohs can be also observed in the stroke subject, i.e., ED and FD CMCohs shifted to the central region, and TRI and BIC CMCohs shifted to the contralesional (right) hemisphere. Figure 2 represents the topography features of 64% stroke subjects (9/14) and 70% unimpaired subjects (7/10) in this work.

**Table:** 

**Figures**	**Scheme**		**ED**	**FD**	**TRI**	**BIC**
(B)	Stroke 20%Ex	Peak Value	0.0287	0.0276	0.0343	0.0394
		Frequency	27.54	27.54	18.75	22.85
		Channel	C_3_	C_z_	C_4_	C_4_
(C)	Control 20%Ex	Peak Value	0.0346	0.0309	0.0338	0.0391
		Frequency	17.87	21.68	21.09	22.56
		Channel	CP_3_	C_1_	C_1_	FC_3_
(D)	Stroke 20%Fx	Peak Value	0.0319	0.0325	0.0321	0.0297
		Frequency	15.23	20.51	18.46	13.80
		Channel	CP_z_	C_z_	FC_4_	FC_4_
(E)	Control 20%Fx	Peak Value	0.0307	0.0360	0.0323	0.0288
		Frequency	29.88	18.16	21.68	27.83
		Channel	C_3_	FC_3_	FC_3_	FC_3_

**Figure 3:**
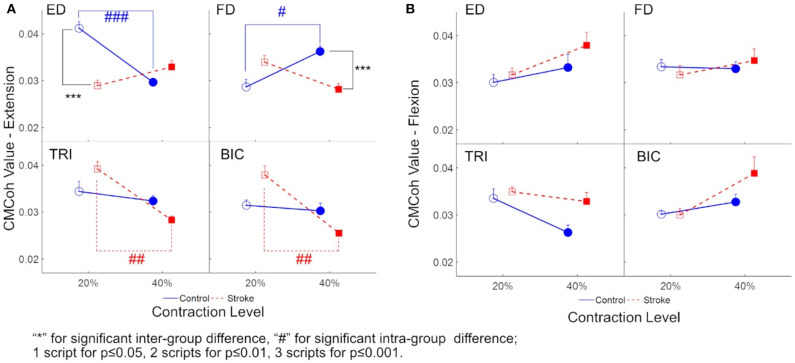
**(A)** The peak CMCoh values of each muscle (i.e., ED, FD, TRI, and BIC) of both groups (the control group: circles; and the stroke group: squares) in 20 and 40% Exs; **(B)** The peak CMCoh values for the muscles of both groups in 20 and 40% Fxs. 1 superscript for *p* ≤ 0.05, 2 superscripts for *p* ≤ 0.01, 3 superscripts for *p* ≤ 0.001. The significant inter-group difference is indicated by ^*^ (independent *t*-test), and # is used to indicate the significant intra-group difference (paired *t*-test).

Figure 3 demonstrates the variations in peak CMCohs of both groups under the two contraction schemes when conducting finger extension (Figure 3A) and flexion (Figure 3B) movements. The following could be observed in Figure 3A during extension tasks: (1) Peak ED CMCohs were significantly lower in the stroke group than in the control group at 20% Ex (*P* < 0.001, effect size [EF]=1.645, independent *t*-test, Table 3); (2) peak FD CMCohs were significantly lower in the stroke group than in the control group at 40% Ex (*P* < 0.001, EF = 1.098, independent *t*-test, Table 3); (3) there was no significant intergroup difference between the stroke subjects and unimpaired controls in terms of peak TRI and BIC CMCohs observed during finger extension movements at both 20 and 40% Ex schemes. Figure 3A also demonstrates the following in terms of the intragroup comparison during extension tasks: (1) Peak ED CMCohs at 20% Ex were significantly higher (*P* < 0.001, EF = 1.969, paired *t*-test, Table 3) than those at 40% Ex for the unimpaired control subjects; (2) peak FD CMCohs at 20% Ex were significantly lower than those at 40% Ex (*P* < 0.05, EF = 1.057, paired *t*-test, Table 3) in the control group; (3) there was no significant change in the peak TRI or BIC CMCohs between the 20 and the 40% Ex schemes in the control group; (4) by contrast, the stroke group showed significantly higher peak TRI and BIC CMCohs at 20% Ex than those at 40% Ex (TRI: *P* < 0.01, EF = 1.324, paired *t*-test, Table 3; BIC: *P* < 0.01, EF = 1.366, paired *t*-test, Table 3); (5) there was no significant intragroup difference in peak ED or FD CMCohs between the two extension schemes for the stroke subjects. Figure 3B demonstrates that there was no significant intergroup difference between stroke and unimpaired control subject and no significant intragroup difference between 20 and 40% Fx schemes in the peak CMCohs as observed. All the statistical results pertaining to the values of peak CMCohs are summarized in Table 3, including the means and 95% confidence intervals, together with the paired *t*-test probabilities and EFs with respect to contraction levels, and independence *t*-test probabilities and EFs with respect to the group.

**Table 3:** The means and 95% confidence intervals of the CMCohs in each muscle during different contraction schemes with paired *t*-test probabilities between the two contraction levels and the independent *t*-test probabilities between stroke and unimpaired control groups.

		**Mean (95% Confidential Interval)** **× 10**^****−2****^		***P*-value (Effect size)**
**CMCoh**	**Group**	**20% Ex**	**40% Ex**	**Intra-group *T*-test**
**ED**	Stroke	2.89 (2.39–3.40)	3.29 (2.56–3.67)	0.410 (0.490)
	Control	4.12 (3.60–4.62)	2.96 (2.65–3.28)	0.001^###^ (1.969)
Inter-group *T*-test	*P*-value (Effect size)	0.001*** (1.645)	0.511 (0.482)	
**FD**	Stroke	3.04 (2.46–3.39)	2.77 (2.49–3.02)	0.113 (0.623)
	Control	2.86 (2.27–3.45)	3.62 (3.15–4.09)	0.035^#^ (1.057)
Inter-group *T*-test	*P*-value (Effect size)	0.564 (0.607)	0.001*** (1.098)	
**TRI**	Stroke	3.91 (3.23–4.52)	2.88 (2.52–3.24)	0.010^##^ (1.324)
	Control	3.44 (2.65–4.23)	3.24 (2.81–3.65)	0.666 (0.234)
Inter-group *T*-test	*P*-value (Effect size)	0.315 (0.454)	0.111 (0.725)	
**BIC**	Stroke	3.79 (2.78–4.72)	2.55 (2.30–2.80)	0.009^##^ (1.366)
	Control	3.14 (2.74–3.55)	3.03 (2.39–3.66)	0.784 (0.168)
Inter-group *T*-test	*P*-value (Effect size)	0.105 (0.674)	0.089 (0.755)	
**CMCoh**	**Group**	**20% Fx**	**40% Fx**	**Intra-Group** ***T*****-test**
**ED**	Stroke	3.17 (2.58–3.76)	3.79 (2.65–4.92)	0.141 (0.442)
	Control	3.00 (2.40–3.62)	3.32 (2.34–4.30)	0.537 (0.277)
Inter-group *T*-test	*P*-value (Effect size)	0.687 (0.186)	0.514 (0.293)	
**FD**	Stroke	3.17 (2.37–3.96)	3.47 (2.64–4.47)	0.400 (0.196)
	Control	3.33 (2.61–4.07)	3.29 (2.73–3.86)	0.914 (0.054)
Inter-group *T*-test	*P*-value (Effect size)	0.737 (0.120)	0.764 (0.158)	
**TRI**	Stroke	3.49 (3.07–3.91)	3.29 (2.51–4.05)	0.475 (0.186)
	Control	3.33 (2.60–4.10)	2.62 (2.06–3.19)	0.094 (0.798)
Inter-group *T*-test	*P*-value (Effect size)	0.704 (0.169)	0.168 (0.668)	
**BIC**	Stroke	3.00 (2.45–3.55)	3.88 (2.44–5.32)	0.169 (0.472)
	Control	3.02 (2.54–3.49)	3.28 (2.69–3.86)	0.502 (0.422)
Inter-group *T*-test	*P*-value (Effect size)	0.962 (0.071)	0.450 (0.354)	

1 superscript for p ≤ 0.05, 2 superscripts for p ≤ 0.01, 3 superscripts for p ≤ 0.001. The significant inter-group difference is indicated by ^*^ (independent t-test), and # is used to indicate the significant intra-group difference (paired t-test).

### EMG Parameters

**Figure 4:**
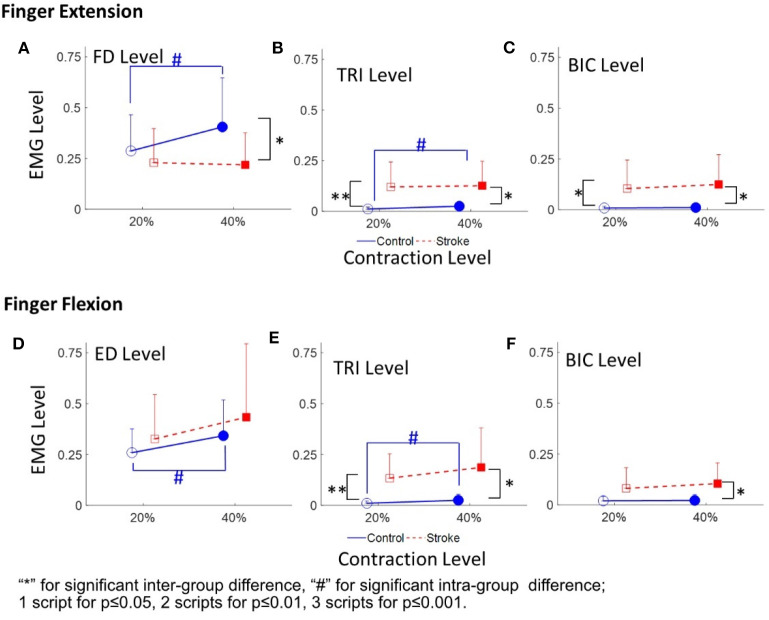
The EMG activation levels of FD **(A)**, TRI **(B)**, and BIC **(C)** in 20 and 40% Exs, and the EMG activation levels of ED **(D)**, TRI **(E)**, and BIC **(F)** in 20 and 40% Fxs, for the two groups. 1 superscript for *p* ≤ 0.05, 2 superscripts for *p* ≤ 0.01, 3 superscripts for *p* ≤ 0.001. The significant inter-group difference is indicated by ^*^ (independent *t*-test), and # is used to indicate the significant intra-group difference (paired *t*-test).

Figure 4 demonstrates the normalized EMG activation levels observed in the four muscles during different contraction schemes. In each motion scheme, the EMG activation level of the agonist muscle, i.e., ED or FD, was maintained at around 0.2 or 0.4 (i.e. the 20 and 40% target level), respectively, during the finger extensions and flexions.

Figures 4A–C shows the normalized EMG activation levels of FD, TRI, and BIC during finger extension. Significant intergroup differences were observed in the FD at 40% Ex (*P* < 0.05, EF = 0.915, independent *t*-test, Table 4A) and the TRI and BIC at both 20% Ex (TRI: *P* < 0.01, EF = 1.254, independent *t*-test; BIC: *P* < 0.05, EF = 0.964, independent *t*-test, Table 4A) and 40% Ex (TRI: *P* < 0.05, EF = 1.162, independent *t*-test; BIC: *P* < 0.05, EF = 1.085, independent *t*-test, Table 4A). Significantly lower FD but higher TRI and BIC activation levels were observed in the stroke group than those observed in the control group. Significant intragroup differences between the 20% Ex and 40% Ex contraction levels were observed only in the control group in the FD and TRI muscles (FD: *P* < 0.05, EF = 0.557, paired *t*-test; TRI: *P* < 0.05, EF = 0.955, paired *t*-test, Table 4A).

**Table 4:** The mean and 95% confidence intervals of EMG parameters during finger extension and flexion, with the intra-group paired *t*-test probabilities between the two contraction levels and the inter-group independent *t*-test probabilities.

		**Mean (95% Confidential Interval)** **× 10**^****−2****^		***P*-value (Effect size)**
**EMG Level**	**Group**	**20% Ex**	**40% Ex**	**Intra-group *T*-test**
**(A) THE EMG ACTIVATION LEVEL**				
**FD**	Stroke	22.92 (12.33–33.50)	21.81 (11.88–31.74)	0.722 (0.068)
	Control	24.25 (15.28–33.21)	40.50 (21.89–59.10)	0.021^#^ (0.557)
Inter-group *T*-test	*P*-value (Effect size)	0.447 (0.334)	0.043* (0.915)	
**TRI**	Stroke	12.01 (4.291–19.73)	12.58 (4.942–20.23)	0.777 (0.048)
	Control	1.110 (0.406–1.803)	2.508 (1.119–3.823)	0.012^#^(0.955)
Inter-group *T*-test	*P*-value (Effect size)	0.010** (1.254)	0.014* (1.162)	
**BIC**	Stroke	9.919 (0.960–18.87)	10.85 (1.704–20.01)	0.213 (0.138)
	Control	0.844 (0.212–1.476)	1.058 (0.210–1.904)	0.430 (0.208)
Inter-group *T*-test	*P*-value (Effect size)	0.048* (0.964)	0.038* (1.085)	
**EMG Level**	**Group**	**20% Fx**	**40% Fx**	**Intra-group** ***T*****-test**
**ED**	Stroke	32.67 (18.94–46.39)	43.36 (17.93–68.78)	0.083 (0.359)
	Control	25.93 (17.46–34.40)	34.35 (21.65–46.84)	0.033^#^ (0.558)
Inter-group *T*-test	*P*-value (Effect size)	0.369 (0.385)	0.484 (0.321)	
**TRI**	Stroke	13.39 (5.878–20.91)	18.68 (6.500–30.87)	0.096 (0.329)
	Control	1.059 (0.216–1.903)	2.522 (0.417–4.623)	0.039^#^ (0.656)
Inter-group *T*-test	*P*-value (Effect size)	0.004** (1.453)	0.014* (1.166)	
**BIC**	Stroke	8.121 (1.771–14.46)	10.47 (4.077–16.85)	0.180 (0.232)
	Control	2.013 (0.419–3.607)	2.195 (0.900–4.297)	0.802 (0.071)
Inter-group *T*-test	*P*-value (Effect size)	0.062 (0.837)	0.017* (1.108)	
**CI**	**Group**	**20% Ex**	**40% Ex**	**Intra-group** ***T*****-test**
**(B) THE CI BETWEEN MUSCLES**				
**ED-FD**	Stroke	14.76 (9.026–20.50)	16.37 (9.897–22.83)	0.291 (0.165)
	Control	16.93 (14.35–19.51)	30.32 (22.40–38.22)	0.001^###^ (1.655)
Inter-group *T*-test	*P*-value (Effect size)	0.503 (0.312)	0.007** (1.319)	
**ED-BIC**	Stroke	7.858 (1.803–13.91)	9.714 (2.926–16.50)	0.082 (0.182)
	Control	0.844 (0.212–1.476)	1.058 (0.210–1.905)	0.430 (0.208)
Inter-group *T*-test	*P*-value (Effect size)	0.027* (1.026)	0.017* (1.128)	
**ED-TRI**	Stroke	9.645 (3.764–15.53)	11.20 (4.851–17.54)	0.394 (0.159)
	Control	1.110 (0.405–1.801)	2.509 (1.194–3.824)	0.012^#^ (0.956)
Inter-group *T*-test	*P*-value (Effect size)	0.008** (1.285)	0.012* (1.198)	
**FD-BIC**	Stroke	8.110 (1.363–14.85)	9.682 (1.876–17.49)	0.143 (0.136)
	Control	0.799 (0.240–1.358)	1.058 (0.210–1.905)	0.322 (0.263)
Inter-group *T*-test	*P*-value (Effect size)	0.036* (0.962)	0.033* (0.978)	
**FD-TRI**	Stroke	8.970 (1.928–16.01)	9.886 (2.982–16.79)	0.414 (0.083)
	Control	1.100 (0.406–1.796)	2.508 (1.193–3.822)	0.012^#^ (0.958)
Inter-group *T*-test	*P*-value (Effect size)	0.032* (0.99)	0.004** (0.938)	
**BIC-TRI**	Stroke	6.174 (0.366–11.98)	7.619 (1.126–14.11)	0.192 (0.147)
	Control	0.310 (0.101–0.519)	0.527 (0.274–0.780)	0.060 (0.693)
Inter-group *T*-test	*P*-value (Effect size)	0.048* (0.898)	0.035* (0.971)	
**CI**	**Group**	**20% Fx**	**40% Fx**	**Intra-Group** ***T-*****test**
**ED-FD**	Stroke	18.54 (11.31–25.78)	25.09 (15.94–34.23)	0.006^##^ (0.499)
	Control	18.34 (14.54–22.16)	26.69 (20.19–33.20)	0.004^##^ (1.128)
Inter-group *T*-test	*P*-value (Effect size)	0.957 (0.023)	0.770 (0.133)	
**ED-BIC**	Stroke	5.246 (1.515–8.977)	7.949 (2.552–13.34)	0.095 (0.366)
	Control	2.005 (0.417–3.592)	2.185 (0.103–4.267)	0.803 (0.071)
Inter-group *T*-test	*P*-value (Effect size)	0.099 (0.725)	0.064 (0.902)	
**ED-TRI**	Stroke	8.664 (3.925–13.40)	11.55 (5.060–18.06)	0.165 (0.533)
	Control	1.059 (0.214–1.903)	2.519 (0.415–4.623)	0.039^#^ (0.656)
Inter-group *T*-test	*P*-value (Effect size)	0.005** (1.488)	0.028* (1.012)	
**FD-BIC**	Stroke	6.260 (1.927–10.59)	9.015 (4.041–13.98)	0.010^#^ (0.371)
	Control	2.001 (0.424–3.580)	2.193 (0.096–4.291)	0.790 (0.076)
Inter-group *T*-test	*P*-value (Effect size)	0.062 (0.834)	0.014* (1.147)	
**FD-TRI**	Stroke	10.80 (5.364–16.24)	14.25 (7.410–21.09)	0.016^#^ (0.351)
	Control	1.056 (0.213–1.898)	2.520 (0.417–4.624)	0.039^#^ (0.658)
Inter-group *T*-test	*P*-value (Effect size)	0.002** (1.581)	0.003** (1.473)	
**BIC-TRI**	Stroke	5.090 (1.277–8.903)	6.863 (2.920–10.81)	0.177 (0.288)
	Control	0.617 (0.164–1.071)	1.622 (0.025–3.270)	0.109 (0.609)
Inter-group *T*-test	*P*-value (Effect size)	0.026* (1.038)	0.016* (1.113)	

1 superscript for p ≤ 0.05, 2 superscripts for p ≤ 0.01, 3 superscripts for p ≤ 0.001. The significant inter-group difference is indicated by ^*^ (independent t-test), and # is used to indicate the significant intra-group difference (paired t-test).

Figures 4D–F present the normalized EMG activation levels of ED, TRI, and BIC during flexion motion tasks. Significant intergroup differences were observed in the BIC at 40% Fx (*P* < 0.05, EF = 1.108, independent *t*-test, Table 4A) and in the TRI at both 20% Fx (*P* < 0.01, EF = 1.453, independent *t*-test, Table 4A) and 40% Fx (*P* < 0.05, EF = 1.166, independent *t*-test, Table 4A). Higher TRI and BIC activation levels were also observed in the stroke group than those in the control group. Significant intragroup differences were observed only in the control group in the ED and TRI muscles between the 20 and 40% Ex contraction levels (ED: *P* < 0.05, EF = 0.558, paired *t*-test; TRI: *P* < 0.05, EF = 0.656, paired *t*-test, Table 4A).

**Figure 5:**
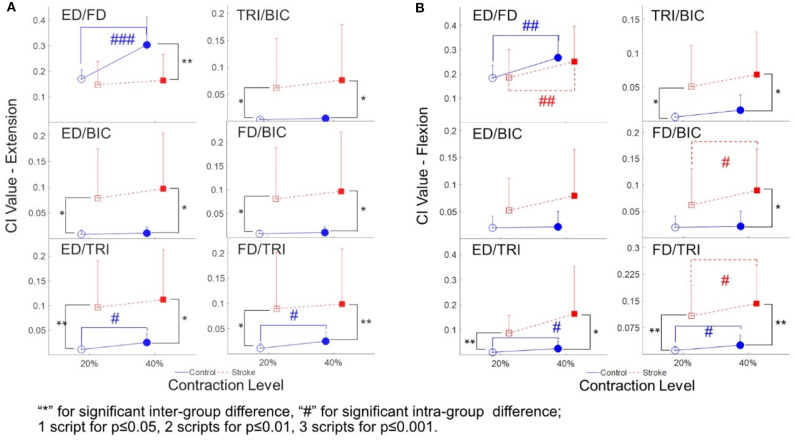
The CI between the muscles of both stroke subjects (squares) and unimpaired subjects (circles): **(A)** The CI values of different muscle pairs during the extensions; **(B)** the CI values of different muscle pairs during the flexions. 1 superscript for *p* ≤ 0.05, 2 superscripts for *p* ≤ 0.01, 3 superscripts for *p* ≤ 0.001. The significant inter-group difference is indicated by ^*^ (independent *t*-test), and # is used to indicate the significant intra-group difference (paired *t*-test).

The varied patterns observed in the CI values between each pair of muscles are illustrated in Figure 5. During the extension schemes, significant intergroup differences in CIs between the stroke and unimpaired subjects could be observed in the ED-BIC, ED-TRI, TRI-BIC, FD-BIC and FD-TRI muscle pairs at both 20 and 40% Ex (*P* < 0.05, independent *t*-test, Table 4B). While ED-FD only demonstrated significant intergroup difference at 40% Ex (*P* < 0.001, EF = 1.319, independent *t*-test, Table 4B). Significant intragroup differences were observed only in the control group in the ED-FD (*P* < 0.05, EF = 1.655, paired *t*-test, Table 4B), ED-TRI (*P* < 0.05, EF = 0.956, paired *t*-test, Table 4B), and FD-TRI (*P* < 0.05, EF = 0.958, paired *t*-test, Table 4B) muscle pairs. During the flexion schemes, significant intergroup differences were observed in the ED-TRI at both 20% Fx (*P* < 0.01, EF = 1.488, independent *t*-test, Table 4B) and 40% Fx (*P* < 0.05, EF = 1.012, independent *t*-test, Table 4B), FD-BIC at 40% Fx (*P* < 0.05, EF = 1.147, independent *t*-test, Table 4B), FD-TRI at both 20% Fx (*P* < 0.01, EF = 1.581, independent *t*-test, Table 4B) and 40% Fx (*P* < 0.01, EF = 1.473, independent *t*-test, Table 4B), and TRI-BIC at both 20% Fx (*P* < 0.05, EF = 1.038, independent *t*-test, Table 4B) and 40% Fx (*P* < 0.05, EF = 1.113, independent *t*-test, Table 4B). The intragroup differences between 20 and 40% Fx could be observed in the ED-FD (*P* < 0.05, EF = 0.499, paired *t*-test, Table 4B), FD-BIC (*P* < 0.05, EF = 0.371, paired *t*-test, Table 4B) and FD-TRI (*P* < 0.05, EF = 0.351, paired *t*-test, Table 4B) muscle pairs in stroke subjects, while significant intragroup changes in unimpaired subjects were observed in the ED-FD (*P* < 0.01, EF = 1.128, paired *t*-test, Table 4B), ED-TRI (*P* < 0.05, EF = 0.656, paired *t*-test, Table 4B), FD-TRI (*P* < 0.05, EF = 0.658, paired *t*-test, Table 4B) muscle pairs.

Table 4 summarizes the statistical analysis of the EMG parameters, including the means and 95% confidence intervals, together with the paired *t*-test probabilities and EFs with respect to contraction levels, and independence *t*-test probabilities and EFs with respect to the group.

## Discussion

The participants in this study performed isometric finger extension and flexion movements at constant and moderate contraction levels. The values of ED, FD, TRI, and BIC CMCohs were significant (*CMCoh* (σ)>0.0170) in both stroke and unimpaired control groups, which suggested the existence of corticomuscular functional coupling from the motor cortex to the muscles in the four distal UE motion schemes (i.e., 20, 40% Ex, 20, and 40% Fx). Although the CMCoh could represent a descending control from the motor cortex to the muscles, high-value CMCohs did not indicate increased muscular output; rather, an increase in cortical control of the movement precision, e.g., either excitatory or inhibitory (39, 43). Therefore, the EMG parameters from the muscles were used to provide supplementary information to the significant CMCohs to support the identification of insights into the exact function of the coupling in the results. Distinct central rhythms and descending control of the UE muscles in both groups were observed in this study.

### Finger Extension

The peak ED CMCohs of the unimpaired control group were significantly higher at 20% Ex than those observed at 40% Ex (Figure 3A). This was indicative of a higher assertion of cortical efforts in a more precise and agile motion control, which was consistent with the fact that most subjects exhibited more difficulties (higher degree of fluctuation of error) during the 30 s contraction tasks within the 20% scheme than they showed within the 40% scheme. We estimated the contraction accuracy for the two groups by evaluating the root-mean-square-error (RMSE) between the real-time contraction level of a target muscle (i.e., *EMGc* in Equation 1) in both extension and flexion and the target contraction level (i.e., 20 or 40% Ex and Fx). It was observed that during 20% Ex and Fx, the stroke (0.056 ± 0.033, mean ± standard deviation) demonstrated larger (*P* = 0.012, *t*-test) RMSE than the control (0.021 ± 0.022). However, there was no difference (*P* = 0.319, *t*-test) in RMSE between the two (the stroke: 0.051 ± 0.031; the control: 0.035 ± 0.037) during 40% Ex and Fx. The RMSEs showed no difference between the 20 and 40% contraction levels for the stroke group (*P* = 0.614, paired *t*-test). However, stroke participants reported verbally the experienced difficulties in controlling 20% contractions. Previous studies have found similar findings showing a correlation between motor cortical activation and the level of motion difficulties (44–47). The peak TRI and BIC CMCohs exhibited no significant changes between 20 and 40% Ex in the unimpaired subjects, implying uniform cortical effort over the two proximal muscles in the finger contractions.

The ED CMCoh patterns observed in the unimpaired group were not replicated in the ED of stroke subjects; however, they were observed across other UE muscles, especially the proximal UE muscles (i.e., TRI and BIC), which exhibited higher CMCohs than those of the FD during the 20% Ex. It demonstrated the cortical deviation of “learned-disuse” pattern after long-term loss of distal muscle functions and relevant proximal compensatory contractions (48, 49). The result was consistent with the EMG activation level results (Figures 4A–C) and the CI values (Figure 5A): Figure 4A demonstrated comparable FD EMG activation levels of both groups at 20% Ex and significant lower values in stroke group at 40% Ex, while Figures 4B,C illustrated significantly higher TRI and BIC activation levels at both 20 and 40% Ex in the stroke group than those in the control group. Besides, Figure 5A demonstrated that the co-contraction patterns between proximal and distal muscles of the chronic stroke patients, with significant higher CI values of ED-TRI, ED-BIC, FD-TRI, FD-BIC, and TRI-BIC at both 20 and 40% Ex, were different from those of the unimpaired controls. The EMG results illustrated that the proximal UE muscles were mainly activated by the brain during the finger extensions even at a lower contraction level and showed markable lower motion independence of the distal fingers in patients with chronic stroke. The shift of peak CMCoh values in Figure 2 further indicated that the activation of contralesional sensorimotor cortex mainly contributed to such brain-induced compensatory pattern in a “learned-disuse” behavioral change'. The neurophysiological basis could be associated the capability of the neurons and neuron aggregates to adapt to the brain lesion (50): As a result of damage to the brain neuron axon, its stump is extended to the target issues or neuron cells to facilitate the creation of new synapses (51). At the same time, normal axons in the proximity of the injured region grow and extend to the target neurons (52). Previous studies involving fMRI have predominantly reported a shift in corticomotor activations from the ipsilesional side to the contralesional side in chronic stroke patients (53, 54). However, there is a lack of evidence relating to the impact that ipsilateral/contralesional corticospinal connections have on the distal muscle control during hand movements after stroke (55–57). The results of the current study (Figure 2) suggested that the similar cortical location shift was mainly related to the compensatory effects of the proximal UE muscles. The synaptic pruning and recreation (synaptogenesis) of neurons potentially stimulate the proximal muscles to perform a compensatory function.

The peak ED CMCohs of the stroke group increased from the low-level contraction to the high-level task. This could be due to the muscle weakness of ED (41) and the fact that stroke patients need to recruit more cortical effort when performing a higher-level contraction task. Unlike the ED muscle, the peak FD CMCohs of the unimpaired control group were lower at 20% Ex than that at 40% Ex. This was because FD is the antagonist muscle to the ED muscle; as such, to maintain joint stability within the wrist at a higher extension contraction level (58, 59), the subjects needed to increase the level of cortical effort to perform FD contractions correspondingly. By contrast, the peak FD CMCohs of the stroke group decreased from low-level to high-level finger extensions and the values were significantly lower than those of the unimpaired control group. It raised a possibility that the weakened antagonism of FD muscle after the stroke as shown in the EMG parameters, i.e., lower FD activation level and lower ED-FD CI than the control, was subsequent to the central functional loss of ED, rather than an initiative weakness in the flexor muscle.

### Finger Flexion

Larsen et al. reported a reduction in CMCoh immediately following stroke and indicated that CMCoh did not appear significantly in the early recovery of hand function (25). In the current study, we found significant peak CMCoh (*CMCoh* (σ)>0.0170) of all UE muscles in chronic stroke patients during both 20 and 40% Fx schemes. This suggested that the corticomuscular coupling has been reestablished during the chronic stage of stroke (19, 26). However, the reconstructed pattern could be different from that of the CMCohs observed among the movements of the unimpaired subjects. Even though the peak FD and ED CMCohs of the chronic stroke subjects during the flexion schemes have reached similar levels to those observed among the unimpaired subjects (Figure 3), the peak CMCohs of the distal muscles have shifted to the central region (Figure 2). This could be attributed to the activation of bilateral cortico-reticulospinal connections, as indicated in the study on primates performed by Soteropoulos et al. (60), which found that pontomedullary reticular formation contributed to the control of finger motions, especially those related to slow and fine movements.

In the unimpaired control group, the increment in peak BIC CMCohs and the reduction of peak TRI CMCohs observed from low-level to high-level contractions were associated with the stable EMG activation levels of both proximal UE muscles. The significant peak CMCohs of TRI and BIC could be related to the inhibition of the motions from the proximal muscles during the distal movements; i.e., the CMCoh of TRI was for the inhibition of the antagonist extensor and CMCoh of BIC was for the synergistic flexor during the finger flexion movements in the control (52, 61, 62).

Despite the fact that no significant intra-group and inter-group difference in peak TRI and BIC CMCohs was found, significant differences in the EMG parameters were observed between the stroke group and the unimpaired control groups as shown in Figures 4D–F, 5B. Figure 4D demonstrated significant increase of ED activation level from 20 to 40% Fx in the unimpaired group, while no similar variation was found in the stroke group. It is reasonable that most stroke patients had the extensor weakness and was used to the “learned-disuse” pattern. Figure 4E demonstrated that the TRI activation levels were significantly higher in the stroke group than those in the control group during both 20 and 40% Fx, and Figure 4F demonstrated that BIC activation levels were significantly higher in the stroke group than those in the control group during 40% Fx. Furthermore, Figure 5B showed that the CI values of TRI-BIC, ED-TRI, FD-TRI at both 20 and 40% Fx as well as the FD-BIC at 20% Fx were also significantly higher in the stroke group. The results indicated that the proximal muscles were abnormally activated during the finger flexions in chronic stroke. Similar to our previous work (6, 7, 35), the results as shown in Figures 4D–F illustrated synchronic co-contractions of TRI and BIC during the finger flexions and suggested that the proximal muscles compensated for the distal flexors after stroke. Besides, in the finger flexion movements of the current study, we found that the peak TRI and BIC CMCohs shifted from the ipsilesional side to the contralesional side in chronic stroke patients, similar to the patterns observed in their finger extension exercise (Figure 2). Similar findings were reported by Chen et al. in chronic stroke patients with flexion synergy, while the increment of CMCohs of proximal flexors was expressed as a result of increased shared neural drive to both proximal and distal UE flexors (26). These may reflect a possibility that the original inhibition corticomuscular coupling has changed to a facilitation function to activate the proximal compensatory movements for the finger flexion. More investigations are required to confirm the mechanism of neuromuscular coupling after a proximal shift to the contralesional side.

In this study, the stroke participants were chronic strokes with moderate motor impairments measured by clinical behavioral assessments (Table 1B). In the chronic stage, behavioral compensation was usually developed. Previous fMRI studies on both animals and humans suggested that increased neural compensation at the cortical level could be interhemispheric for behavioral restorations after stroke (63–65). Carey and Wilkins also detected a shift of corticomotor activations from the ipsilesional to the contralesional in chronic stroke patients (53, 54). The mechanism for the cortical center shift after stroke as suggested by Christian et al. (50) and Chechik et al. (51) was related to the neural plastic strategies by recruiting resources from contralesional (i.e., interhemispheric) and additional intrahemispheric areas. Similar shifts of peak CMCohs were captured in the stroke participants in this study (Figures 2B,D). It suggested that the cortical compensation happened in chronic stroke with moderate motor impairments. Furthermore, the CMCohs shifts were mainly related to the proximal muscles in the stroke group, i.e., TRI and BIC shifted to the contralesional side with higher intensities (Figures 2B,D), compared to the shifts in the FD and ED (ipsilesional and center areas). It suggested that the post-stroke compensation facilitated more neuroplastic activities on the proximal muscles at the cortical level than the distal finger muscles. The behavioral changes in relation to this proximal compensation were represented by the significant increases in EMG levels (Figure 4) and CI values (Figure 5) related to the TRI and BIC detected peripherally.

## Limitations

The main limitation of this study is the small sample size. Despite the relatively small populations recruited, we observed consistent results on the corticomuscular variation patterns demonstrated by the stroke patients by both CMCoh and EMG parameters. More investigations with larger scales will be conducted in the future to investigate the mechanism of neuromuscular coupling after a proximal shift to the contralesional side, and also to track the CMCoh variations from early/subacute stroke to the chronic period.

## Conclusion

Neuromuscular coupling during dynamic muscular contraction as measured by CMCoh and EMG parameters were adopted in this study to investigate the corticomuscular coordinating pattern of post-stroke compensatory activities from the proximal UE when performing distal motions at the affected side. The results suggested that the proximal UE compensatory action of the distal finger in chronic stroke patients was cortically derived, and the TRI and BIC were mainly activated from the contralesional side. This study confirmed the cortical activation shift in chronic stroke reported by other functional neuroimaging studies and further demonstrated that the cortical shift was concentrated within the proximal UE muscles as opposed to the distal agonist muscles. Specifically, the stroke patients needed to recruit more cortical effort of ED to conduct higher-level contraction. In contrast, the corticomuscular coupling to the FD during finger flexion was comparable in terms of the intensity and pattern in the peak CMCoh of the stroke subjects and the unimpaired subjects. However, the CMCoh results showed that, similar to that of the ED, the neural drive to FD shifted to the central region in chronic stroke subjects.

## Data Availability Statement

The raw data supporting the conclusions of this article will be made available by the authors, without undue reservation, to any qualified researcher.

## Ethics Statement

The study was carried out in accordance with the human ethic guidelines of the Human Subjects Ethics Subcommittee of the Hong Kong Polytechnic University. All participants recruited in the study gave written informed consent in accordance with the Declaration of Helsinki before the start of the measurements. The patients/participants provided their written informed consent to participate in this study.

## Author Contributions

ZG and QQ equally contributed in the experiment design, data collection and analysis, and manuscript drafting. KW and HZ contributed in the system design and maintenance. YH contributed in the subject recruitment and data collection. YZ contributed in the data analysis. XH conceived the study and coordinated the whole project, including the experiment design, system design, and manuscript drafting.

## Conflict of Interest

The authors declare that the research was conducted in the absence of any commercial or financial relationships that could be construed as a potential conflict of interest.

## References

[1] Langhorne P Bernhardt J Kwakkel G. Stroke rehabilitation. Lancet. (2011) 377:1693–702. 10.1016/S0140-6736(11)60325-5 21571152

[2] Dobkin BH. Rehabilitation after stroke. N Engl J Med. (2005) 352:1677–84. 10.1056/NEJMcp043511 15843670 PMC4106469

[3] Kong K-H Chua KS Lee J. Recovery of upper limb dexterity in patients more than 1 year after stroke: frequency, clinical correlates and predictors. NeuroRehabilitation. (2011) 28:105–11. 10.3233/NRE-2011-0639 21447911

[4] Kwakkel G Wagenaar RC Kollen BJ Lankhorst GJ. Predicting disability in stroke—a critical review of the literature. Age Ageing. (1996) 25:479–89. 10.1093/ageing/25.6.479 9003886

[5] Good DC Bettermann K Reichwein RK. Stroke rehabilitation. Continuum. (2011) 17:545–67. 10.1212/01.CON.0000399072.61943.38 22810867

[6] Nam C Rong W Li W Xie Y Hu X Zheng Y. The effects of upper-limb training assisted with an electromyography-driven neuromuscular electrical stimulation robotic hand on chronic stroke. Front Neurol. (2017) 8:679. 10.3389/fneur.2017.00679 29312116 PMC5735084

[7] Qian Q Nam C Guo Z Huang Y Hu X Ng SC. Distal versus proximal - an investigation on different supportive strategies by robots for upper limb rehabilitation after stroke: a randomized controlled trial. J Neuroeng Rehabil. (2019) 16:64. 10.1186/s12984-019-0537-5 31159822 PMC6545723

[8] Jones TA. Motor compensation and its effects on neural reorganization after stroke. Nat Rev Neurosci. (2017) 18:267–80. 10.1038/nrn.2017.26 28331232 PMC6289262

[9] Pineiro R Pendlebury S Johansen-Berg H Matthews P. Functional MRI detects posterior shifts in primary sensorimotor cortex activation after stroke: evidence of local adaptive reorganization? Stroke. (2001) 32:1134–9. 10.1161/01.STR.32.5.1134 11340222

[10] Calautti C Leroy F Guincestre J-Y Baron J-C. Displacement of primary sensorimotor cortex activation after subcortical stroke: a longitudinal PET study with clinical correlation. Neuroimage. (2003) 19:1650–4. 10.1016/S1053-8119(03)00205-2 12948719

[11] Platz T van Kaick S Möller L Freund S Winter T Kim IH. Impairment–oriented training and adaptive motor cortex reorganisation after stroke: a fTMS study. J Neurol. (2005) 252:1363–71. 10.1007/s00415-005-0868-y 15965585

[12] Mima T Hallett M. Electroencephalographic analysis of cortico-muscular coherence: reference effect, volume conduction and generator mechanism. Clin Neurophysiol. (1999) 110:1892–9. 10.1016/S1388-2457(99)00238-2 10576484

[13] Mima T Matsuoka T Hallett M. Information flow from the sensorimotor cortex to muscle in humans. Clin Neurophysiol. (2001) 112:122–6. 10.1016/S1388-2457(00)00515-0 11137669

[14] Mima T Steger J Schulman AE Gerloff C Hallett M. Electroencephalographic measurement of motor cortex control of muscle activity in humans. Clin Neurophysiol. (2000) 111:326–37. 10.1016/S1388-2457(99)00229-1 10680569

[15] Salenius S Portin K Kajola M Salmelin R Hari R. Cortical control of human motoneuron firing during isometric contraction. J Neurophysiol. (1997) 77:3401–5. 10.1152/jn.1997.77.6.3401 9212286

[16] Gerloff C Uenishi N Nagamine T Kunieda T Hallett M Shibasaki H. Cortical activation during fast repetitive finger movements in humans: steady-state movement-related magnetic fields and their cortical generators. Electroencephalogr Clin Neurophysiol Electromyogr Motor Control. (1998) 109:444–53. 10.1016/S0924-980X(98)00045-9 9851302

[17] Brown P Salenius S Rothwell JC Hari R. Cortical correlate of the Piper rhythm in humans. J Neurophysiol. (1998) 80:2911–7. 10.1152/jn.1998.80.6.2911 9862895

[18] Govindan RB Raethjen J Kopper F Claussen JC Deuschl G Estimation of time delay by coherence analysis. Phys A Stat Mech Appl. (2005) 350:277–95. 10.1016/j.physa.2004.11.043

[19] Meng F Tong K Chan S Wong W Lui K Tang K. Cerebral plasticity after subcortical stroke as revealed by cortico-muscular coherence. IEEE Transac Neural Syst Rehabil Eng. (2009) 17:234–43. 10.1109/TNSRE.2008.2006209 19273041

[20] Mima T Toma K Koshy B Hallett M. Coherence between cortical and muscular activities after subcortical stroke. Stroke. (2001) 32:2597–601. 10.1161/hs1101.098764 11692023

[21] Weiller C Chollet F Friston KJ Wise RJS Frackowiak RSJ. Functional reorganization of the brain in recovery from striatocapsular infarction in man. Ann Neurol. (1992) 31:463–72. 10.1002/ana.410310502 1596081

[22] Weder B Knorr U Herzog H Nebeling B Kleinschmidt A Huang Y. Tactile exploration of shape after subcortical ischaemic infarction studied with PET. Brain. (1994) 117:593–605. 10.1093/brain/117.3.593 8032868

[23] Seitz RJ Huang Y Knorr U Tellmann L Herzog H Freund HJ. Large-scale plasticity of the human motor cortex. Neuroreport. (1995) 6:742–4. 10.1097/00001756-199503270-00009 7605938

[24] Fang Y Daly JJ Sun J Hvorat K Fredrickson E Pundik S. Functional corticomuscular connection during reaching is weakened following stroke. Clin Neurophysiol. (2009) 120:994–1002. 10.1016/j.clinph.2009.02.173 19362515 PMC2680928

[25] Larsen LH Zibrandtsen IC Wienecke T Kjaer TW Christensen MS Nielsen JB. Corticomuscular coherence in the acute and subacute phase after stroke. Clin Neurophysiol. (2017) 128:2217–26. 10.1016/j.clinph.2017.08.033 28987993

[26] Chen X Xie P Zhang Y Chen Y Cheng S Zhang L. Abnormal functional corticomuscular coupling after stroke. NeuroImage Clin. (2018) 19:147–59. 10.1016/j.nicl.2018.04.004 30035012 PMC6051472

[27] Xu R Wang Y Wang K Zhang S He C Ming D. Increased corticomuscular coherence and brain activation immediately after short-term neuromuscular electrical stimulation. Front Neurol. (2018) 9:886. 10.3389/fneur.2018.00886 30405518 PMC6206169

[28] Rong W Li W Pang M Hu J Wei X Yang B. A Neuromuscular Electrical Stimulation (NMES) and robot hybrid system for multi-joint coordinated upper limb rehabilitation after stroke. J Neuroeng Rehabil. (2017) 14:34. 10.1186/s12984-017-0245-y 28446181 PMC5406922

[29] Folstein MF Folstein SE McHugh PR. “Mini-mental state”: a practical method for grading the cognitive state of patients for the clinician. J Psychiatr Res. (1975) 12:189–98. 10.1016/0022-3956(75)90026-6 1202204

[30] Fugl-Meyer AR Jääskö L Leyman I Olsson S Steglind S. The post-stroke hemiplegic patient. 1. a method for evaluation of physical performance. Scand J Rehabil Med. (1975) 7:13–31. 1135616

[31] Bohannon RW Smith MB. Interrater reliability of a modified ashworth scale of muscle spasticity. Phys Ther. (1987) 67:206–7. 10.1093/ptj/67.2.206 3809245

[32] Hu XL Tong RKY Ho NSK Xue JJ Rong W Li LSW. Wrist rehabilitation assisted by an electromyography-driven neuromuscular electrical stimulation robot after stroke. Neurorehabil Neural Repair. (2015) 29:767–76. 10.1177/1545968314565510 25549656

[33] von Carlowitz-Ghori K Bayraktaroglu Z Hohlefeld FU Losch F Curio G Nikulin VV Corticomuscular coherence in acute and chronic stroke. Clin Neurophysiol. (2014) 125:1182–91. 10.1055/s-0034-1371233 24315544

[34] Sheng Y Liu J Liu H. Corticomuscular coherence and its applications: a review. Front Hum Neurosci. (2019) 13:100. 10.3389/fnhum.2019.00100 30949041 PMC6435838

[35] Qian Q Hu X Lai Q Ng SC Zheng Y Poon W. Early stroke rehabilitation of the upper limb assisted with an electromyography-driven neuromuscular electrical stimulation-robotic arm. Front Neurol. (2017) 8:447. 10.3389/fneur.2017.00447 28928706 PMC5591334

[36] Zheng Y Gao L Wang G Wang Y Yang Z Wang X. The influence of unilateral contraction of hand muscles on the contralateral corticomuscular coherence during bimanual motor tasks. Neuropsychologia. (2016) 85:199–207. 10.1016/j.neuropsychologia.2016.03.028 27018484

[37] Zheng Y Peng Y Xu G Li L Wang J. Using corticomuscular coherence to reflect function recovery of paretic upper limb after stroke: a case study. Front Neurol. (2018) 8:728. 10.3389/fneur.2017.00728 29375467 PMC5767581

[38] Tecchio F Porcaro C Zappasodi F Pesenti A Ercolani M Rossini PM. Cortical short-term fatigue effects assessed via rhythmic brain–muscle coherence. Exp Brain Res. (2006) 174:144–51. 10.1007/s00221-006-0432-8 16604318

[39] Divekar NV John LR. Neurophysiological, behavioural and perceptual differences between wrist flexion and extension related to sensorimotor monitoring as shown by corticomuscular coherence. Clin Neurophysiol. (2013) 124:136–47. 10.1016/j.clinph.2012.07.019 22959414

[40] Lou X Xiao S Qi Y Hu X Wang Y Zheng X. Corticomuscular coherence analysis on hand movement distinction for active rehabilitation. Comput Math Methods Med. (2013) 2013:10. 10.1155/2013/908591 23690885 PMC3652035

[41] McClelland VM Cvetkovic Z Mills KR. Rectification of the EMG is an unnecessary and inappropriate step in the calculation of corticomuscular coherence. J Neurosci Methods. (2012) 205:190–201. 10.1016/j.jneumeth.2011.11.001 22120690

[42] Rosenberg JR Amjad AM Breeze P Brillinger DR Halliday DM. The Fourier approach to the identification of functional coupling between neuronal spike trains. Prog Biophys Mol Biol. (1989) 53:1–31. 10.1016/0079-6107(89)90004-7 2682781

[43] Schulz H Übelacker T Keil J Müller N Weisz N Now i am ready—now i am not: the influence of Pre-TMS oscillations and corticomuscular coherence on motor-evoked potentials. Cerebral Cortex. (2013) 24:1708–19. 10.1093/cercor/bht024 23395847

[44] Boroojerdi B Ziemann U Chen R Bütefisch CM Cohen LG. Mechanisms underlying human motor system plasticity. Muscle Nerve. (2001) 24:602–13. 10.1002/mus.1045 11317269

[45] Calautti C Leroy F Guincestre J-Y Baron J-C. Dynamics of motor network overactivation after striatocapsular stroke. Stroke. (2001) 32:2534–42. 10.1161/hs1101.097401 11692013

[46] Hallett M. Plasticity of the human motor cortex and recovery from stroke. Brain Res Rev. (2001) 36:169–74. 10.1016/S0165-0173(01)00092-3 11690613

[47] Wilcox T Hawkins LB Hirshkowitz A Boas DA. Cortical activation to object shape and speed of motion during the first year. Neuroimage. (2014) 99:129–41. 10.1016/j.neuroimage.2014.04.082 24821531 PMC4228933

[48] Bobath B Adult Hemiplegia: Evaluation and Treatment. Oxford: Butterworth-Heinemann (1990).

[49] Kwakkel G Wagenaar RC. Effect of duration of upper- and lower-extremity rehabilitation sessions and walking speed on recovery of interlimb coordination in hemiplegic gait. Phys Ther. (2002) 82:432–48. 10.1093/ptj/82.5.432 11991797

[50] Christian KM Poulos AM Thompson RF Learning andmemory: basic principles and model systems. In: Kwakkel G Cohen L Selzer M Miller R Clarke S, editors. Textbook of Neural Repair and Rehabilitation: Volume 1: Neural Repair and Plasticity, 2 ed. Cambridge: Cambridge University Press (2014). p. 22–35.

[51] Chechik G Meilijson I Ruppin E. Synaptic pruning in development: a computational account. Neural Comput. (1998) 10:1759–77. 10.1162/089976698300017124 9744896

[52] Huttenlocher PR Dabholkar AS. Regional differences in synaptogenesis in human cerebral cortex. J Comp Neurol. (1997) 387:167–78. 9336221 10.1002/(sici)1096-9861(19971020)387:2<167::aid-cne1>3.0.co;2-z

[53] Carey JR Kimberley TJ Lewis SM Auerbach EJ Dorsey L Rundquist P. Analysis of fMRI and finger tracking training in subjects with chronic stroke. Brain. (2002) 125:773–88. 10.1093/brain/awf091 11912111

[54] Wilkins KB Owen M Ingo C Carmona C Dewald JPA Yao J. Neural plasticity in moderate to severe chronic stroke following a device-assisted task-specific arm/hand intervention. Front Neurol. (2017) 8:284. 10.1093/med/9780199689903.003.0010 28659863 PMC5469871

[55] Werhahn KJ Conforto AB Kadom N Hallett M Cohen LG. Contribution of the ipsilateral motor cortex to recovery after chronic stroke. Ann Neurol. (2003) 54:464–72. 10.1002/ana.10686 14520658

[56] Soteropoulos DS Edgley SA Baker SN. Lack of evidence for direct corticospinal contributions to control of the ipsilateral forelimb in monkey. J Neurosci. (2011) 31:11208–19. 10.1523/JNEUROSCI.0257-11.2011 21813682 PMC3183456

[57] Zaaimi B Edgley SA Soteropoulos DS Baker SN. Changes in descending motor pathway connectivity after corticospinal tract lesion in macaque monkey. Brain. (2012) 135:2277–89. 10.1093/brain/aws115 22581799 PMC3381720

[58] Forner-Cordero A Levin O Li Y Swinnen SP. Posture control and complex arm coordination: analysis of multijoint coordinative movements and stability of stance. J Mot Behav. (2007) 39:215–26. 10.3200/JMBR.39.3.215-226 17550873

[59] Raghavan P. Upper limb motor impairment after stroke. Phys Med Rehabil Clin. (2015) 26:599–610. 10.1016/j.pmr.2015.06.008 26522900 PMC4844548

[60] Soteropoulos DS Williams ER Baker SN. Cells in the monkey ponto-medullary reticular formation modulate their activity with slow finger movements. J Physiol. (2012) 590:4011–27. 10.1113/jphysiol.2011.225169 22641776 PMC3476645

[61] Kozlowski DA James DC Schallert T. Use-dependent exaggeration of neuronal injury after unilateral sensorimotor cortex lesions. J Neurosci. (1996) 16:4776–86. 10.1523/JNEUROSCI.16-15-04776.1996 8764664 PMC6579010

[62] Frost SB Barbay S Friel KM Plautz EJ Nudo RJ. Reorganization of remote cortical regions after ischemic brain injury: a potential substrate for stroke recovery. J Neurophysiol. (2003) 89:3205–14. 10.1152/jn.01143.2002 12783955

[63] He BJ Snyder AZ Vincent JL Epstein A Shulman GL Corbetta M. Breakdown of functional connectivity in frontoparietal networks underlies behavioral deficits in spatial neglect. Neuron. (2007) 53:905–18. 10.1016/j.neuron.2007.02.013 17359924

[64] Carter AR Astafiev SV Lang CE Connor LT Rengachary J Strube MJ. Resting interhemispheric functional magnetic resonance imaging connectivity predicts performance after stroke. Ann Neurol. (2010) 67:365–75. 10.1002/ana.21905 20373348 PMC2927671

[65] van Meer MPA van der Marel K Wang K Otte WM El Bouazati S Roeling TAP. Recovery of sensorimotor function after experimental stroke correlates with restoration of resting-state interhemispheric functional connectivity. J Neurosci. (2010) 30:3964. 10.1523/JNEUROSCI.5709-09.2010 20237267 PMC6632290

